# Cephalopod genomics: A plan of strategies and organization

**DOI:** 10.4056/sigs.3136559

**Published:** 2012-09-26

**Authors:** Caroline B. Albertin, Laure Bonnaud, C. Titus Brown, Wendy J. Crookes-Goodson, Rute R. da Fonseca, Carlo Di Cristo, Brian P. Dilkes, Eric Edsinger-Gonzales, Robert M. Freeman, Roger T. Hanlon, Kristen M. Koenig, Annie R. Lindgren, Mark Q. Martindale, Patrick Minx, Leonid L. Moroz, Marie-Therese Nödl, Spencer V. Nyholm, Atsushi Ogura, Judit R. Pungor, Joshua J. C. Rosenthal, Erich M. Schwarz, Shuichi Shigeno, Jan M. Strugnell, Tim Wollesen, Guojie Zhang, Clifton W. Ragsdale

**Affiliations:** 1 Department of Organismal Biology and Anatomy, University of Chicago, Chicago, IL, USA; 2 Muséum National d'Histoire Naturelle, Université Paris Diderot, Sorbonne Paris Cité, Paris, France; 3 Departments of Computer Science and Engineering, and Microbiology and Molecular Genetics, Michigan State University, East Lansing, MI, USA; 4 Soft Matter Materials Branch, Materials and Manufacturing Directorate, Air Force Research Laboratory, Wright-Patterson AFB, OH, USA; 5 Natural History Museum of Denmark, University of Copenhagen, Copenhagen, Denmark; 6 Department of Biological, Geological and Environmental Sciences, University of Sannio, Benevento, Italy; 7 Department of Horticulture and Landscape Architecture, Purdue University, West Lafayette, IN, USA; 8 Department of Molecular and Cell Biology, University of California at Berkeley, Berkeley, CA, USA; 9 Department of Systems Biology, Harvard Medical School, Boston, MA, USA; 10 Marine Biological Laboratory, Woods Hole, MA, USA; 11 Institute of Cell and Molecular Biology, University of Texas at Austin, Austin, TX, USA; 12 Department of Biology, Portland State University, Portland, OR, USA; 13 Kewalo Marine Laboratory, University of Hawaii, Honolulu, HI, USA; 14 The Genome Institute, Washington University School of Medicine, St. Louis, MO, USA; 15 Whitney Laboratory for Marine Bioscience, University of Florida, St. Augustine, FL, USA; 16 Department of Theoretical Biology, University of Vienna, Vienna, Austria; 17 Department of Molecular and Cell Biology, University of Connecticut, Storrs, CT, USA; 18 Institute for Genome Research, University of Tokushima, Tokushima, Japan; 19 Hopkins Marine Station of Stanford University, Pacific Grove, CA, USA; 20 Institute of Neurobiology, University of Puerto Rico Medical Sciences Campus, San Juan, PR, USA; 21 Department of Molecular Biology and Genetics, Cornell University, Ithaca, NY, USA; 22 Japan Agency for Marine-Earth Science and Technology, Yokosuka, Japan; 23 Department of Genetics, La Trobe University, Bundoora, Victoria, Australia; 24 Department of Integrative Zoology, University of Vienna, Vienna, Austria; 25 BGI-Shenzhen, Shenzhen, China; 26 Department of Neurobiology, University of Chicago, Chicago, IL, USA

## Abstract

The Cephalopod Sequencing Consortium (CephSeq Consortium) was established at a NESCent Catalysis Group Meeting, “Paths to Cephalopod Genomics- Strategies, Choices, Organization,” held in Durham, North Carolina, USA on May 24-27, 2012. Twenty-eight participants representing nine countries (Austria, Australia, China, Denmark, France, Italy, Japan, Spain and the USA) met to address the pressing need for genome sequencing of cephalopod mollusks. This group, drawn from cephalopod biologists, neuroscientists, developmental and evolutionary biologists, materials scientists, bioinformaticians and researchers active in sequencing, assembling and annotating genomes, agreed on a set of cephalopod species of particular importance for initial sequencing and developed strategies and an organization (CephSeq Consortium) to promote this sequencing. The conclusions and recommendations of this meeting are described in this white paper.

## Cephalopods

Cephalopods (octopus, squid, cuttlefish, *Nautilus*) have captured the imagination of scientists and the general public since Aristotle. These predatory creatures are an ancient group, known from at least the Late Cambrian and today comprising more than 700 species [1,2]. Cephalopods range in size from the pygmy squids (thumbnail-sized adults) to the colossal and giant squids (18 meters in total length), which are the largest known invertebrates. Cephalopods are believed to be among the most “advanced” invertebrates, having evolved large, highly differentiated brains, a sophisticated set of sensory organs that includes vertebrate-like eyes, and fast jet-propelled locomotion [3]. The neuroendocrine and heart-blood vascular systems of cephalopods have long been recognized for their complexity and similarity to those found in vertebrates [4-6]. A particularly striking trait of cephalopods is that they are masters of rapid adaptive coloration, having the ability to change quickly the texture, pattern, color and brightness of their skin. Dynamic camouflage helps the animals evade detection by predators and approach prey with stealth; the same systems produce signals for communication with conspecifics [3]. The remarkable morphological and physiological innovations of cephalopods provide the scientific community with a tremendous opportunity for insight into mechanisms of evolutionary convergence and innovation in structure and function.

Cephalopods have diversified to inhabit all oceans of the world, from benthic to pelagic zones, from intertidal areas to the deep sea, and from the polar regions to the tropics. They share the “behavioral space” in their many marine habitats with teleost fishes and marine mammals [7], placing them in some of the most competitive ecohabitats on Earth. Cephalopods are ecologically important for the central position they play in trophic predator-prey relationships; they are a primary food source for marine mammals and for many harvested fish species. Their importance in the food web is often underestimated, but they constitute a crucial element in coastal ecosystem equilibrium. Moreover, cephalopods themselves are the target of large commercial fisheries worldwide, with an annual harvest of two million metric tons of squid alone [8].

Cephalopod biological research has a long history involving a wide range of experimental paradigms, the best known of which is the work on squid giant axon physiology that led to Nobel Prize awards for Alan Hodgkin and Andrew Huxley. Also prominent are the extensive investigations by J.Z. Young, Brian Boycott, Martin Wells and colleagues into cephalopod brain and behavior, with a particular focus on the sophisticated learning and memory systems of the octopus [9]. Cephalopod biology has recently become relevant to the field of biomimetic research, particularly for robotics and materials science [10,11]. There are likely to be many new areas of cephalopod-based research. For example, cephalopods immobilize prey organisms with toxins, some of which are very poisonous to humans [1]. Study of such toxins may serve to identify new biomedically valuable reagents [12].

Cephalopods are mollusks, which show a greater variety of forms than do any other extant animal phylum. Even within the Mollusca, cephalopods display a remarkable level of modification in body plan organization. Particularly notable among the soft-bodied (coleoid) cephalopods are the reduction or loss of the shell, the adaptation of the mantle for locomotion and respiration, and the modification of the ventral molluscan foot into arms [2]. These innovations are undoubtedly tightly linked to the selective pressures from the loss of the shell and the development of a “high-performance” nervous system. The cephalopod lineage, and its origins from a monoplacophoran-like molluscan ancestor [2,13], thus represents a deeply attractive model for understanding the acquisition of novelty through evolutionary time.

All of these areas of cephalopod biology, from neuronal function at the cellular and systems levels to cephalopod population dynamics to the evolution of gene regulatory elements mediating body plan variation, would benefit greatly from the molecular insight that high-quality cephalopod genomics would provide. Indeed, it is astonishing that, in 2012, with the explosion of genome resources for so many life forms, there is not yet available a single assembled cephalopod genome. The goal of the NESCent meeting and this white paper is to provide organizational mechanisms for cephalopod biology to move from the pre-genomic to the post-genomic age.

## Genomics

Genomic and transcriptomic sequencing will greatly aid the biological study of cephalopods. A sequenced genome produces a comprehensive list of genes, and contains the regulatory blueprint dictating their expression [14]. Sequenced transcriptomes reveal the expression levels of gene sets for different cells, tissues and organs at different developmental stages and under different physiological states [15,16]. Resequencing individuals of a genome-enabled species offers unprecedented datasets that can be applied to long-standing questions in population genetics, disease, and the characterization of species of commercial importance where there may be little *a priori* genetic knowledge [17,18]. Comparative genomics has revolutionized and stabilized our understanding of the evolutionary relationships among organisms throughout the Tree of Life, both living and recently extinct [19,20]. Sequence data have also advanced novel areas of research, such as nanotechnology, biomaterials and synthetic biology [21-23].

The most obvious benefit of cephalopod genomics will be to individual laboratories already studying cephalopod biology. With a full inventory and complete sequences for known genes of interest, laboratories can study gene function much more rapidly and thoroughly. In addition, with a near-complete inventory of protein-coding and non-coding RNA genes, these researchers can assess a much larger set of candidate genes for function in their biological processes of interest.

The greater benefits may come, however, to biological researchers outside the existing cephalopod field. Until very recently, genome-scale analyses of biological processes have favored the sequencing of two out of the three major divisions of bilateral animals [24]: deuterostomes (primarily vertebrates, with an expanding study of other chordates and selected non-chordates such as sea urchins and hemichordates) and ecdysozoans (from which the model organisms *Drosophila melanogaster* and *Caenorhabditis elegans* both come). In contrast, there has been far less genomic analysis of lophotrochozoans, with genomes published for only a handful of organisms, including three trematode parasitic worms and one oyster [25-29]. The genes and gene networks regulating the independent evolution of the host of highly derived features displayed in cephalopods are unknown, making comparative analyses of these phenomena at the level of gene function and regulation impossible. Sequencing of cephalopods would do more than expand our knowledge of genome organization within lophotrochozoans. With genomic data, researchers currently studying molecular evolution of complex metazoans would be able to investigate cephalopods as a new, independent instance of such evolution.

The genomes of cephalopods are known to be larger and more repeat-rich than many previously sequenced metazoan genomes [30]. With newly developed methods for sequencing and assembly [31,32], these genomes are now more tractable than they would have been even a few years ago. Indeed, the likely challenges of cephalopod genomics will prove an important test of these emerging technologies.

Genomic data will allow analyses of cephalopod molecular biology that have, until now, not been considered by the cephalopod community. Detailed studies of the genomes of mammals, flies, and nematodes have revealed unanticipated mechanisms of gene regulation: microRNAs-first characterized through nematode genetics and then shown to be ubiquitous [33]; epigenetic modification of the genome-first documented through the genetics of *Drosophila* position-effect variegation and then mechanistically clarified by studies in many species, including mammals [34,35]; and long non-coding RNAs-initially identified in mammals (*Xist*, *H19)* and flies (BX-C) and subsequently found to be pervasive [36,37]. The extent to which gene and protein expression in mollusks is regulated by the mechanisms identified in mouse, fruit fly, and nematode is unknown, but one striking example is provided by RNA editing. This regulatory process for protein diversification was initially described in mammals, but now appears to be much more widely employed in cephalopods than in vertebrates [38,39]. It is possible that deeper genomic studies of mollusks, and in particular cephalopods, will reveal additional, as yet undiscovered mechanisms of animal gene regulation.

Another promising arena of research that may benefit from cephalopod genomics is the global analysis of protein-coding gene families [40], which has to date been strongly biased towards deuterostomes and ecdysozoans. Proteins in these two groups feature extremely well characterized domains as well as domains that remain completely obscure and are typically described as "Domain of Unknown Function" [41]. Cephalopod genomics can be expected to enrich our knowledge of such protein domain modules. Moreover, study of cephalopods will also almost undoubtedly expand the pool of protein domains, as it has already done in the identification of the reflectin protein family [11].

## Choices of cephalopod species for genomic sequencing

Within the Mollusca, cephalopods diverged from a monoplacophoran-like ancestor over 500 million years ago, later branching into the extant clades Nautiloidea (*Nautilus* and *Allonautilus*) and Coleoidea (squid, cuttlefish and octopus) [2,42-44]. The CephSeq Consortium has come together with the intention of using strategic genomic and transcriptomic sequencing of key cephalopod species to address previously unanswerable questions about this group. Taking into account the challenges of cephalopod genome sequencing, as well as the necessity to address nodal taxa, we have identified a set of species on which to focus our initial efforts. Selected species have been chosen based on the curiosity of their biological features as well as the possible advantages of their practical use. These species also cover ecologically diverse life histories, representing benthic, nectobenthic and nectonic animals.

Cephalopods are animals with advanced cognitive skills and a complex repertoire of behavioral abilities [3,45]. Their brains are comparable both in size and complexity with those of vertebrates, and have been the focus of a number of studies on the neurobiology of behavior [46]. In particular, they have served as models for the cellular and systems circuitry of learning and memory [4,9]. Historically, *Octopus vulgaris* has been a key species for this work through studies of anatomy [9], behavior following lesions and brain stimulation [3,4,47] and cellular neurophysiology [48,49]. *O*. *vulgaris* has also served as an attractive model for neuroendocrine studies in invertebrates [5,50].

Recently, *Octopus bimaculoides* (California Two-spot Octopus) has emerged as a model system for cephalopod biology. The large size of *O. bimaculoides* eggs grants unique access to early embryonic stages, making this species a prime candidate for future genetic and developmental studies. The hardiness, ready availability in the United States and easy husbandry of adult *O*. *bimaculoides* [51] add to the appeal of this model species.

The deadly venom of blue-ringed octopus *Hapalochlaena maculosa* makes this species of interest for study of the evolution and regulation of toxicity within octopods [1].

Comparative studies of these octopus species would illuminate the bases of both their shared characteristics as well as those of their divergent features. Additionally, these species have essentially non-overlapping geographic distributions, providing animal accessibility to cephalopod researchers globally.

Within the decapodiforms, *Sepia* and *Loligo* are the most studied genera. Historically, *Sepia officinalis* has been a key cephalopod for neurobiological research, and is a critical species in global fisheries. *S. officinalis* possesses a complex chromatophore network for countershading, camouflage and communication [3,52,53]. Its internal calcified shell supplies buoyancy and the effect of global climate changes on this structure has become a focus of recent study [54,55]. *S. officinalis* is emerging as a particularly versatile model organism in eco-evo-devo studies [56]. As a practical matter, *S. officinalis* eggs are voluminous, and easily collected, maintained and reared in the laboratory [57]. The morphological events in *S. officinalis* embryogenesis are well described in the literature [58-61].

*Loligo*, and particularly its giant fiber system, has served as the fundamental basis for our understanding of nerve impulse conduction. The giant synapse system has recently been employed as a biomedical model of neurological disease [62]. *Loligo* is one of the most important groups for cephalopod fisheries in the North Atlantic [8]. *Loligo pealeii* is the premier experimental species of the loliginids, with not only an extensive publication base [63], but also annual availability at the Marine Biological Laboratory (Woods Hole, MA).

*Euprymna scolopes* is a unique cephalopod model organism because of its well-described symbiotic relationship with the luminescent bacterium *Vibrio fischeri*. This important biomedical model has been employed to study the mechanisms of host colonization and symbiont specificity, host/microbe cell-cell signaling, and innate immunity [64-67]. *Euprymna scolopes*’ short life cycle and small egg size also make it an attractive choice for developmental studies in culture [68,69]. In 2005, the *V. fischeri* genome was sequenced [70]; having access to the host genome would allow this field to advance rapidly.

Pygmy squids (*Idiosepius*) have one of the smallest genomes among cephalopods (2.1 Gb), making them strong candidates for assembly and annotation [30]. Their small body size and exceptionally short life cycle also distinguish these cephalopods as possible model organisms [71].

The giant squid *Architeuthis dux* serves to represent deep-sea cephalopods. Little is known about the species of *Architeuthis*. *Architeuthis* is globally distributed and a recent analysis of the complete mitogenomes of multiple giant squid worldwide showed no detectable phylogenetic structure on the mitochondrial level and an exceptionally low level of nucleotide diversity, suggesting that there is only one global species of giant squid [72]. A nuclear reference genome for *Architeuthis* would clarify the population genetics of this species and provide critical information for comparative studies across cephalopods.

*Nautilus*, the cephalopod “living fossil”, is a representative of a phylogenetically unique branch of the cephalopods, the nautiloids. *Nautilus* possesses many presumably ancestral anatomical features not shared with other cephalopods, including pinhole eyes, rhinophores for odor detection, an external shell, and numerous tentacles, all without suckers [73]. Comparative genomic studies employing *Nautilus* would highlight the genetic bases of these divergent features.

## Sequencing strategy

Cephalopod genomes are large, complex and full of repeats. Sequencing and assembly may be technically very challenging. Below we recommend what, with the current state of hardware and software, would be excellent approaches to tackling cephalopod genomes. Researchers in the CephSeq Consortium will undoubtedly choose varying combinations of approaches for their specific projects. In any event, with rapid changes in the underlying technologies for sequencing, assembly and annotation, this series of technical recommendations will need to be revisited on a regular basis, and should be viewed as the snapshot it is of a particular moment (May 2012) in a rapidly advancing field.

Our recommendation for the initial approach to genome sequencing of cephalopods is to use a proven low-cost short-read sequencing approach (Illumina HiSeq with long-insert mate pairs). The current best practices for initial assembly of complex (≥1 Gb) eukaryotic genomes involve a mixture of high read coverage derived from short insert libraries (300-2000 bp) and high clone-coverage of longer insert (5-10 kb) and fosmid jump libraries (or mate-pair libraries). In this approach, approximately 45× coverage from the smaller insert libraries and 45× coverage from a 5-kb insert library would be produced for each taxon. In addition, 5× read coverage would be generated for 10-kb insert size libraries. For increasing genomic contiguity and long-range scaffolding, 40-kb fosmid jump libraries at 1× genomic coverage should be added for the ten pioneer cephalopod genomes (see Table 1). These methods have been tested and were successful in the sequencing of the 2.4 Gb giant panda [74] and the *de novo* assembly of the 3.2 Gb human genome with ALLPATHS-LG [75]. Additional approaches, such as sequence-based genetic mapping to bridge the gap between scaffolds and chromosomes and emerging long-read single molecule technologies (PacBio RS), could also be employed.

**Table 1:** Cephalopod species proposed for initial sequencing efforts.

Species	Estimated genome size	Current sequencing coverage	Geographic distribution	Lifestyle juvenile/adult	Research importance
*O. vulgaris*	2.5-5 Gb	46×	world-wide	planktonic/ benthic	classic model for brain and behavior, fisheries science
*O. bimaculoides*	3.2 Gb	50×	California, Mexico	benthic	emerging model for development and behavior, fisheries science
*H. maculosa*	4.5 Gb	10×	Indo-Pacific	benthic	toxicity
*S. officinalis*	4.5 Gb	-	East Atlantic- Mediterranean	nectobenthic	classic model for behavior and development, fisheries science
*L. pealeii*	2.7 Gb	-	Northwest Atlantic	nectonic	cellular neurobiology, fisheries science
*E. scolopes*	3.7 Gb	-	Hawaii	nectobenthic	animal-bacterial symbiosis, model for development
*I. paradoxus*	2.1 Gb	80×	Japan	nectobenthic	model for development, small genome size
*I. notoides*	-	50×	Australia	nectobenthic	model for development, small genome size
*A. dux*	4.5 Gb	60×	world-wide	nectonic	largest body size
*N. pompilius*	2.8-4.2 Gb	10×	Indo-Pacific	nectonic	“living fossil”, outgroup to coleoid cephalopods

Initial efforts in cephalopod genomics, as well as more mature efforts in other molluscan genomes (*Aplysia, Biomphalaria, Lottia*), have identified many challenges in generating useful genomic assemblies. Many specific taxa were discussed at the NESCent meeting, and several collaborative projects have been initiated. For example, two species of *Octopus* will soon have genomic sequence generated, and two groups plan to sequence the smallest known cephalopod genomes, those of the genus *Idiosepius* (2.1 Gb). There was broad support at the meeting for sequencing *Sepia*, *Loligo*, and *Euprymna*, based on biological significance, research community size and phylogenetic position. Limited genome sequence data from *Sepia officinalis*, *Euprymna scolopes*, *Hapalochlaena maculosa*, *Architeuthis dux* and *Nautilus pompilius* are or will soon be available. Integration of these sequence data will assist with annotation and gene detection by sampling broadly across the phylogeny of cephalopods, with *Nautilus* providing an important outgroup for the coleoid cephalopods. Interpretation of cephalopod-specific genetic novelty and the innovations involved in nervous system specialization would be further assisted by the sequencing of an outgroup such as one from the Monoplacophora. While contiguous and annotated genomes are our ultimate goal, the strong sense of the community is that intermediate assemblies and transcriptome sequencing would be immensely helpful, and ideally would be exchanged prior to publication.

It must be emphasized that all the projects described above are in their infancy and are expected to benefit from the formation of the CephSeq Consortium. Indeed, representatives from each of these cephalopod sequencing efforts participated in the NESCent meeting and agreed to the formation of the Consortium.

Annotation of novel genomes is a complex problem [76]. Efforts at automated annotation of molluscan genomic sequences have demonstrated the challenge facing the future annotation of cephalopod genomes. Long branch lengths within the phylum, the taxonomic distances to well annotated animal genomes, and the relatively low quantity of previous molecular and genetic work in the Mollusca will demand the generation of additional resources to assist and train automated gene detection programs. Of primary importance will be the generation of transcript inventories to identify genes, refine gene models, detect start points and intron-exon boundaries, and train automated gene identification algorithms. Transcriptome data such as those from RNAseq are quick and relatively inexpensive to generate, and will be immensely useful. Systematic sequencing of nervous system tissues and embryonic stages can be combined with relatively early-stage assemblies to generate gene models and exon structures. In addition, pairs of *Octopus* species (*O. vulgaris* and *O. bimaculoides*) and *Idiosepius* species (*I. notoides* and *I. paradoxus*), through comparative sequence analysis, may be critical for annotation.

Annotation efforts are labor-intensive but also offer an opportunity to grow the cephalopod research community and attract outside expertise. For example, domain experts of particular gene families or pathways can be recruited to assist in the description of likely protein function. Bioinformatics researchers interested in the problems of annotation across long phylogenetic distances, the assessment of unique gene families and the evolution of biochemical novelty, and the likely challenges of extensively RNA-edited transcriptomes, will also be enlisted. Finally, annotation provides an outreach opportunity to involve young scientists and K-12 classrooms in cutting-edge scientific discovery on these fascinating organisms.

## Data sharing plan

An important goal of the CephSeq Consortium is to share data rapidly and effectively both within and beyond the Consortium. Data sharing is necessary to foster the broadest possible impact of our sequencing and annotation efforts. This sharing will prove critically important for the cephalopod community. We expect sequence homology within the taxon to be an important foundation for collaboration within the field because cephalopods have evolved many new and unique character features. Sharing data prior to publication could significantly accelerate cephalopod research. However, data sharing policies must also recognize that there is significant publication, funding, and career recognition risks involved in making data available before publication: often the first to publish a particular observation garners the most recognition.

Broad data-sharing agreements such as the Ft. Lauderdale agreement [77] have already been adopted by the international genomics community, and, most significantly, by many large sequencing centers. However, as the sequencing capacity of small collaborations has increased, this type of agreement is an increasingly poor fit for the data being generated. Moreover, for a federated community such as the CephSeq Consortium, with significant international participation by many small groups, enforcement of any agreement is challenging. We believe that an explicit policy should be adopted to protect data generators while creating incentives for the earliest possible sharing of data. An effective policy should also encourage use of cephalopod sequence data beyond the currently defined cephalopod community, while protecting the interests of those generating the data.

We therefore propose to adopt a liberal opt-in data sharing policy, modeled in part on the JGI data usage policy [78], which will support the rapid sharing of sequence data, subject to significant restrictions on certain types of usage. Community members will be encouraged to submit their data, but not required to do so. We plan to provide incentives for this private data sharing by (1) developing a community data and analysis site with a simple set of automated analyses such as contig assembly and RNAseq transcript assembly; (2) offering pre-computed analyses such as homology search across the entire database; and (3) supporting simple investigative analyses such as BLAST and HMMER. We also plan to provide bulk download services in support of analysis and re-analysis of the entire dataset upon mutual agreement between the requesting scientist and the CephSeq Consortium Steering Committee (see below), who will represent the depositing scientists. Collectively, these policies would provide for community engagement and participation with the CephSeq Consortium while protecting the interests of individual contributors, both scientifically and with respect to the Convention on Biological Diversity [79]. Policy details will need to be specified and implementation is subject to funding. Our intent is to build an international community by putting the fewest barriers between the data and potential researchers, while still protecting the data generators.

## The CephSeq Consortium: Mission statement and organization

**Mission Statement:** The vision of the Cephalopod Sequencing Consortium is rapid advancement of cephalopod science into the genomics era, one employing the most modern and efficient methods available and engaging broad international participation by the entire cephalopod scientific community. This vision entails communication and active promotion of sequencing technologies and findings to researchers across a great diversity of fields. Bioinformatics experts initially outside of cephalopod biology will participate with cephalopod researchers in this effort. The Consortium will help facilitate funding endeavors by individuals and groups by providing basic summary documents (*e.g.*, white papers, letters of support) that describe the current state and consensus goals of cephalopod genomics efforts worldwide. In addition to promoting and accelerating scientific progress, the CephSeq Consortium aims to translate the contributions of cephalopod science to society at large by encouraging applied science in fields as diverse as fisheries science, materials science and biomedical research. Education and outreach will be emphasized for broad dissemination of progress in cephalopod genomics at multiple levels, including K-12, undergraduate and graduate students, and the public at large.

**Organizational Structure:** Establishment of a **Steering Committee** was agreed upon at the May 2012 NESCent Catalysis Group Meeting. The composition of the committee was initially set at seven members, with broad international representation of cephalopod biologists, genomicists and bioinformaticians. The Committee will initially meet every 4 months, either in person, or remotely, or both. The Steering Committee is charged with providing international oversight of the community’s activities, fostering the free-flow of information among CephSeq Consortium members (see Data Sharing Plan), promoting collaborations, and ensuring that the CephSeq Consortium remains focused on the Mission Statement objectives set forth above. The Steering Committee will also work to facilitate community-wide efforts to annotate assembled genomes.

The tenure of the Committee will initially be two years, and any and all cephalopod researchers are encouraged to contact the Committee about the changing needs of the community. The inaugural members are: Laure Bonnaud (Univ. Paris, France), C. Titus Brown (Michigan State Univ., USA), Roger Hanlon (Marine Biological Laboratory, USA), Atsushi Ogura (Ochanomizu Univ., Japan), Clifton Ragsdale/Chair (Univ. Chicago, USA), Jan Strugnell (La Trobe Univ., Australia) and Guojie Zhang (BGI, China).

A web site [80] will serve as a point of contact for the worldwide community. An auxiliary site for sharing cephalopod genomic and transcriptomic data is to be established within the next six months (see Data Sharing Plan). The CephSeq Consortium will coordinate internationally with the Cephalopod International Advisory Council (CIAC) [81] and with the newly established CephRes-Associazione Cephalopod Research-ONLUS [82], which is based in Europe.

Workshops will be organized annually to ensure coordinated and cooperative progress in genomics on an international scale. One likely venue for such workshops would be society meetings, such as the annual meeting of the Society for Integrative and Comparative Biology (SICB).

The Steering Committee urges scientists who support the goals of this white paper to join the consortium by signing the white paper and participating in the activities of the consortium.

## Broader impacts

A specific recommendation of this white paper is to compete for a Research Coordination Network (RCN) grant from the NSF. A Cephalopod RCN would facilitate annotation of the cephalopod genomes being produced worldwide, mediate the exchange of emerging technologies that will benefit from genomic resources and accelerate the advent of new areas of research made possible by cephalopod genomics. It would also serve to expand the next generation of cephalopod researchers. Consequently, a central element of a Cephalopod RCN would be short-term laboratory exchanges for undergraduate and graduate students to aid in genome annotation and analysis, to promote education in bioinformatics and cephalopod biology and to foster new collaborations across the cephalopod community.

Cephalopods are important to science, including the fields of cellular neurobiology, learning and memory, neuroethology, biomaterial engineering, animal-microbe interactions, developmental biology, and fundamental molecular biology such as RNA editing. Access to genomic information will greatly facilitate this ongoing research, particularly through gene discovery. Cephalopod genomics will also drive the creation of new areas of investigation, including such biomedically important topics as regeneration and aging [83,84]. Other examples of promising post-genomic cephalopod research include study of the unknown chemosensory systems by which cephalopods monitor their marine environments, and the isolation of cephalopod neurotoxins, which could lead to novel reagents for research and drug-based therapies [12]. Cephalopod genomics will also be important for evolutionary biology, particularly for understanding the great diversity and genomic complexity of the whole molluscan phylum and for probing the emergence of the evolutionary innovations that are represented by cephalopod eyes, large brains and prehensile arms.

Cephalopods are a critical component of marine ecology, are important commercially to the fisheries industry and are an emerging aquaculture taxon. The effects of global warming and marine acidification and hypoxification on cephalopod health and viability are unknown and can only be fully assessed with improved species delineation and a deeper understanding of population dynamics. Specifically, cephalopod genomics will aid our ability to track population migrations and monitor demographic expansions and contractions. This information will in turn directly inform efforts to assess the effects of climate change on cephalopod stocks [85]. Cephalopods are a critical food source and genomic resources can also be expected to help monitor cephalopod overfishing and improve cephalopod aquaculture.

People are fascinated by cephalopods, from *Nautilus* to the octopus to the giant squid. The coupling of genomics to cephalopod biology represents a fusion of two areas of great interest and excitement for the public. This fusion presents a tremendous educational platform, particularly for K-12 students, who can be engaged in the classroom and through the public media. Public outreach about cephalopod genomics will help build support for basic scientific research, including study of marine fauna and ecology, and will add to the public’s understanding of global changes in the biosphere.

## Scientists who have written in support of the white paper

Tom Abrams, University of Maryland School of Medicine, Baltimore, MD, USA

Shelley Adamo, Dalhousie University, Halifax, Nova Scotia, Canada

Louise Allcock, National University of Ireland, Galway, Ireland

Frank E. Anderson, Southern Illinois University, Carbondale, IL, USA

Paul Andrews, St George's, University of London, London, UK

George J. Augustine, Center for Functional Connectomics, KIST, Seoul, Korea

Yann Bassaglia, Univ. Paris-Est Creteil, Creteil, France

Elaine L. Bearer, University of New Mexico Health Sciences Center, Albuquerque, NM, USA

Francisco Bezanilla, University of Chicago, Chicago, IL, USA

Jean Geary Boal, Millersville University, Millersville, PA, USA

Sydney Brenner, Okinawa Institute of Science and Technology, Okinawa, Japan

Euan R. Brown, Heriot-Watt University, Edinburgh, UK

Bernd U. Budelmann, Galveston, TX, USA

Roy Caldwell, University of California, Berkeley, CA, USA

R. Andrew Cameron, California Institute of Technology, Pasadena, CA, USA

David Carlini, American University, Washington, DC, USA

Sean Carroll, University of Wisconsin, Madison, WI, USA

Maria G. Castillo, New Mexico State University, Las Cruces, NM, USA

Thomas W. Cronin, University of Maryland Baltimore County, Baltimore, MD, USA

Joseph A. DeGiorgis, Providence College, Providence, RI, USA

Anna Di Cosmo, University of Naples "FedericoII", Naples, Italy

Ludovic Dickel, University of Caen Lower-Normandy, Caen, France

Casey Dunn, Brown University, Providence, RI, USA

David B. Edelman, Scripps Research Institute, San Diego, CA, USA

David C. Gadsby, The Rockefeller University, New York, NY, USA

Camino Gestal, Institute of Marine Research, Vigo, Spain

M. Thomas P. Gilbert, University of Copenhagen, Copenhagen, Denmark

Ian G. Gleadall, Tohoku University, Sendai, Japan

Takashi Gojobori, National Institute of Genetics, Mishima, Japan

Ralph J. Greenspan, University of California-San Diego, La Jolla, CA, USA

Jeffrey Gross, University of Texas, Austin, TX, USA

Volker Hartenstein, University of California, Los Angeles, CA, USA

Binyamin Hochner, Hebrew University, Jerusalem, Israel

Peter W. H. Holland, University of Oxford, Oxford, UK

Miguel Holmgren, NINDS/National Institutes of Health, Bethesda, MD, USA

Christine L. Huffard, Conservation International, Santa Cruz, CA, USA

Sönke Johnsen, Duke University, Durham, NC, USA

Leonard K. Kaczmarek, Yale University School of Medicine, New Haven, CT, USA

Paul Katz, Georgia State University, Atlanta, GA, USA

William M. Kier, University of North Carolina, Chapel Hill, NC, USA

Michael J. Kuba, Hebrew University, Jerusalem, Israel

Gilles Laurent, Max Planck Institute for Brain Research, Frankfurt, Germany

Mark J. Mandel, Northwestern University Feinberg School of Medicine, Chicago, IL, USA

Felix C. Mark, Alfred Wegener Institute for Polar and Marine Research, Bremerhaven, Germany

I. A. Meinertzhagen, Dalhousie University, Halifax, Nova Scotia, Canada

John B. Messenger, University of Cambridge, Cambridge, UK

Hassan Moustahfid, National Oceanic and Atmospheric Administration, Silver Spring, MD, USA

Michele K. Nishiguchi, New Mexico State, Las Cruces, NM, USA

Mark Norman, Museum Victoria, Melbourne, Australia

Todd H. Oakley, University of California, Santa Barbara, CA, USA

Daniel Osorio, University of Sussex, Brighton, UK

Anna Palumbo, Stazione Zoologica Anton Dohrn, Naples, Italy

Carlos Rosas, Universidad Nacional Autónoma de México, Sisal, Yucatán, México

Greg Rouse, Scripps Institution of Oceanography-UCSD, La Jolla, CA, USA

Michael C. Schmale, University of Miami, Miami, FL, USA

Brad Seibel, University of Rhode Island, Kingston, RI, USA

Paul Shaw, Aberystwyth University, Aberystwyth, UK

Eric V. Stabb, University of Georgia, Athens, GA, USA

Kenneth B. Storey, Carleton University, Ottawa, Ontario, Canada

Nathan Tublitz, University of Oregon, Eugene, OR, USA

Michael Vecchione, Smithsonian Institution, Washington, DC, USA

Janet R. Voight, Field Museum of Natural History, Chicago, IL, USA

Edgar T. Walters, University of Texas Medical School, Houston, TX, USA

Torsten N. Wiesel, The Rockefeller University, New York, NY, USA

James B. Wood, Hawaii Institute of Marine Biology, Kaneohe, HI, USA

Masa-aki Yoshida, National Institute for Genetics, Shizuoka, Japan

Richard E. Young, University of Hawaii, Honolulu, HI, USA

Letizia Zullo, Italian Institute of Technology, Genoa, Italy
